# Conference Report: NIST TUTORIAL/WORKSHOP ON LATTICE GAS AND BOLTZMANN METHODS Gaithersburg, MD August 21-23, 1995

**DOI:** 10.6028/jres.101.068

**Published:** 1996

**Authors:** Nicos S. Martys

**Affiliations:** Building Materials Division, Building and Fire Research Laboratory, National Institute of Standards and Technology, Gaithersburg, MD 20899-0001

## 1. Introduction

The flow of fluids in complex geometries like porous media [1] plays an important role in a wide variety of technological and environmental processes including chromotography, oil recovery, the degradation of building materials and the spread of hazardous wastes in soils. The complexity of such flow processes makes their theoretical and experimental study a great challenge. Recent developments in cellular automata methods such as lattice gas and lattice Boltzmann allow for the accurate solution of the Navier-Stokes equation for multi-phase and multi-component fluid flow [2], [3], [4]. The advantage of these methods over traditional approaches, such as finite difference and finite element methods, is that they naturally accomodate complex boundary conditions such as surface tension forces associated with fluid/surface and fluid/fluid interfaces. They are also capable of describing complex flow problems such as the shearing of non-Newtonian fluids and the phase separation of fluids.

In August 1995, the NIST Center for Theoretical and Computational Material Science (Material Science and Engineering Laboratory) and the NIST Building Materials Division [Building and Fire Research Laboratory (BFRL)] cosponsored a workshop titled *NIST Tutorial/Workshop on Lattice Gas and Lattice Boltzmann Methods*. The organizer was Dr. Nicos S. Martys of the Building Materials Division. The purpose of this workshop was to familiarize NIST scientists and members of industry with the basic ideas of lattice gas and lattice Boltzmann methods and to inform the audience of recent developments in such computational methods.

The workshop was attended by over 50 participants of which approximately 40 % were from industry, 30 % from academia and 30 % from national laboratories.

## 2. Conference Summary

The Lattice Gas/Boltzmann workshop was held over 2 days and divided into two parts. The first part was a tutorial given by Dr. Hudong Chen of Exa Corporation, Cambridge, Massachusetts. Dr. Chen is a world expert on lattice gas and lattice Boltzmann methods having published over forty papers on this relatively new topic. In this tutorial the basic concepts of lattice gas and lattice Boltzmann methods were introduced. The second part of the workshop was devoted to recent developments in, and applications of, these methods. The following sections include representative sample highlights of the workshop.

### 2.1 Modeling of Phase Transtions

Dr. Xiaowen Shan of Los Alamos National Laboratory discussed a recently proposed lattice Boltzmann model for simulating multi-component, multi-phase flows by incorporating long-range interactions into the fluid dynamics. It was shown that the equation of state of the fluid could be altered from an ideal-gasto one that matches any specific functional form. When the equation of state is properly chosen, the fluid system will separate into multiple phases each of which still obeys the Navier Stokes equation. The coexistence curve, density profile across a liquid-gas interface, surface tension, and diffusivity in a multi-component system were obtained analytically for this model.

### 2.2 Chaotic Front Dynamics

Dr. Ray Kapral (Chemical Physics Theory Group, Department of Chemistry, University of Toronto) introduced a lattice gas model describing the mesoscale dynamics of spatially distributed, chemically reacting systems. Results were presented for two examples of such systems. First, chaotic front dynamics that arise from diffusively driven instabilities were described. Second, the dynamics of catalytic oxidation processes on metal surfaces was discussed. In this case, the metal-catalysed oxidation was preceded by the coupling of the reaction kinetics to surface phase reconstruction, and gave rise to a rich variety of pattern formation processes on micrometer to submicrometer length scales. Consequently one could begin to probe fluctuation effects and the breakdown of mean field theories in these systems.

### 2.3 Two-Phase Flow in Porous Rock

John F. Olson in collaboration with Dr. Daniel H. Rothman of the Massachusetts Institute of Technology presented results from lattice gas simulations of two-phase flow through Fontainebleau sandstone. The three-dimensional rock geometry, obtained by x-ray microtomography, was used to investigate complex flows through a natural medium. Results were compared with earlier studies of flow through artificial porous media, and with recent experimental measurements on the same media. These comparisons illuminated the potential and the limitations of these techniques.

### 2.4 Physical Modeling on Cellular Automata Machines

Dr. Norman Margolus of the MIT Lab for Computer Science discussed his group’s involvement in research on large-scale cellular automata (CA) modeling of physical systems. This research has been part of a long term effort to better match computations to the constraints of physics, with the ultimate aim of effectively harnessing the potential of computer hardware that can take advantage of the uniformity, simplicity, and spatial locality of cellular automata computations. Recent developments in dedicated “Cellular Automata Machines” have significantly advanced their ability to efficiently and flexibly model and analyze large-scale cellular automata systems.

### 2.5 Lattice Gas Models of Microemulsion Dynamics

Bruce Boghosian of the Center for Computational Science at Boston University described a lattice gas model of the nonequilibrium dynamics of microemulsions. The model was based on the immiscible lattice gas of Rothman and Keller [3], which is reformulated using a microscopic, particulate description to permit generalization to more complicated interactions. Surfactant particles were then introduced into the model with vector, dipolar interactions. Simulation results and structure-function measurements were presented to demonstrate that the model exhibits the correct phenomenology, including a critical micelle concentration, and emulsion droplet, and lamellar phases.

## 3. Conclusion

The NIST Tutorial/Workshop on Lattice Gas/Boltzmann Methods provided an opportunity for NIST scientists to learn about state-of-art techniques for the modeling of many complex flow problems. The workshop also provided an opportunity for interaction with many of the world leaders in the field of cellular automata modeling of fluids.

There has been ongoing research in the application of lattice Boltzmann methods in the modeling of single and multi-component flow in porous media [5] like building materials (BFRL). Several scientists in different NIST laboratories have also begun to adopt lattice Boltzmann methods to model single and multi-component flow problems such as resin transfer in fiber meshes (Polymers Division) and flow in microgeometries (Computational and Applied Mathematics Laboratory). For further information contact Dr. Nicos S. Martys.
